# A Comparative Analysis of DNA Metabarcoding and Morphological Identification in Diatoms Reveals Similar Patterns of Environmental Response

**DOI:** 10.1002/ece3.72644

**Published:** 2026-02-17

**Authors:** Fernanda Gonzalez‐Saldias, Joan Gomà, Sandra Garcés‐Pastor, Owen S. Wangensteen, Albert Pèlachs, Aaron Pérez‐Haase

**Affiliations:** ^1^ Department of Evolutionary Biology, Ecology, and Environmental Sciences University of Barcelona Barcelona Spain; ^2^ Freshwater Ecology, Hydrology, and Management Research Group (FEHM‐Lab) University of Barcelona Barcelona Spain; ^3^ Institute of Marine Sciences (ICM) CSIC Barcelona Catalonia Spain; ^4^ Geography Department Universitat Autònoma de Barcelona Cerdanyola del Vallès Spain; ^5^ Biodiversity Research Institute (IRBio) University of Barcelona Barcelona Spain

**Keywords:** 18S rRNA, Bacillariophyceae, COI, ecological gradient, mires, pH

## Abstract

Diatoms are widely used as environmental sentinels and are commonly studied through morphological identification. However, this method requires specialist knowledge, which is becoming increasingly scarce, resulting in species identification becoming costly, time‐consuming, and difficult. DNA metabarcoding offers an alternative method to semiautomate some identification processes to circumvent these difficulties, thus minimizing identification costs while providing objective and reliable information. However, discrepancies between marker genes and morphological identification remain unsolved. This study investigated the 66 diatom communities on 26 high mountain mires in the Pyrenees by identifying morphological species and sequencing the Cytochrome c oxidase I (COI) and 18S rRNA markers. We assessed whether morphological and molecular approaches reveal the same patterns of alpha and beta diversity. Our results showed a strong correlation between beta diversity patterns of diatoms obtained by morphological identification and genetic markers. In contrast, alpha diversity calculated through molecular studies underestimated species richness and the Shannon index. The diatom community was chiefly driven by pH, Mg, and temperature, reflecting these patterns in morphological and molecular data. DNA metabarcoding also proved to represent diatom community patterns and the roles of the environmental drivers as clearly as in morphological identifications in high mountain mire diatom communities.

## Graphical abstract

This study compares the use of two universal molecular markers (COI and 18S rRNA) with morphological identification to evaluate patterns in diatom communities from high mountain mires. The results revealed a strong correspondence in beta diversity and environmental response across beta and alpha diversity. However, these molecular markers were found to be inadequate for achieving accurate taxonomic resolution and diversity assessment, as they tend to underestimate alpha diversity, which could potentially lead to misinterpretation of the data.

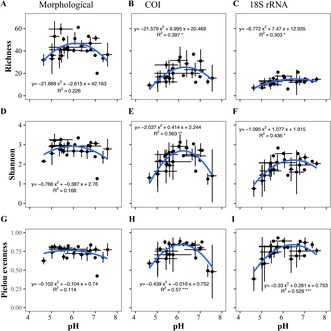

## 1 Introduction

Diatoms are diverse and ubiquitous unicellular algae that play a pivotal role as primary producers in aquatic ecosystems (Mann and Vanormelingen 2013; Round et al. 1990; Zimmermann et al. 2015). Their species distribution is determined by various environmental variables, among which pH (Potapova and Charles 2002), electrical conductivity (Potapova and Charles 2003), temperature, and nutrient availability (Stevenson et al. 2010) stand out. This sensitivity to environmental changes makes diatoms valuable ecological indicators of ecosystem conditions and has often been used to assess eutrophication (Cantonati and Lowe 2014; Rimet 2012).

Traditional methods for identifying and counting diatoms depend on morphological features such as size, shape, and number of striae observed under light or electron microscopy. This dependence on detailed morphology renders the process highly specialized, demanding, and time‐consuming (Kahlert et al. 2009; Mann et al. 2010; Rimet et al. 2018), but important and relevant for ecological studies. Furthermore, cryptic species and phenotypic plasticity within populations complicate accurate species identification (Cox 2014; Mann and Evans 2008). Variations in taxonomists' expertise also contribute to discrepancies in morphological assessments, primarily due to the lack of intercalibration among experts (Vasselon et al. 2019). Consequently, these factors can lead to both underestimation or overestimation of species diversity (Bailet et al. 2020; Kahlert et al. 2009).

To address the challenge of morphological identification, molecular identification through metabarcoding has emerged as a promising alternative (Mann et al. 2010). Using DNA markers, this approach identifies multiple taxa regardless of their life stage (Rimet et al. 2018; Zimmermann et al. 2015). Using universal markers means that multiple taxa can be amplified simultaneously, eliminating the need to separate them in the samples. Such markers provide a good approximation of the maximum biodiversity in environmental DNA samples (Collins et al. 2019; Hadziavdic et al. 2014; Klepke et al. 2022). However, the results obtained from metabarcoding analyses can vary significantly depending on the gene marker used and the completeness and reliability of genetic databases. For instance, notable differences have been observed between the cytochrome oxidase I (COI) and 18S rRNA genes in community representation compared to morphological identification and the number of taxon groups (Kermarrec et al. 2013; Othman et al. 2021; Trobajo et al. 2010).

The COI‐LerayXT marker (313‐bp mitochondrial gene) has been widely used for animal identification due to its robustness and high taxonomic resolution (Mann et al. 2010; Moniz and Kaczmarska 2009; Wangensteen et al. 2018). This DNA region presents a high incidence of nucleotide substitution compared to other genes (Hebert et al. 2003). These characteristics have facilitated its application across various taxa, including protists (Nassonova et al. 2010), macroalgae (Saunders 2005), and diatoms (Evans et al. 2007; Guo et al. 2015; Kermarrec et al. 2013).

Similarly, the 18S rRNA gene has been proposed as a potential barcode marker for identifying eukaryotes, with different regions designated for various groups of organisms (Hadziavdic et al. 2014). For instance, the V1–V2 regions have been utilized for nematodes (Schenk et al. 2020), the V4, V5, and V9 regions for protists (Decelle et al. 2014; Taib et al. 2013), the V7 region for microbial eukaryotic communities (Capo et al. 2021), the V9 region for paleo‐diatoms (Armbrecht et al. 2021), the V4 region for phytoplankton (Esenkulova et al. 2020), and both the V7 and V4 regions for diatoms (Garcés‐Pastor et al. 2019; Zimmermann et al. 2015).

Diatoms, recognized for their sensitivity to environmental variables, serve as excellent models for ecological studies, particularly in diverse aquatic ecosystems like those present in the Pyrenees, which play a crucial role in biodiversity (Rivera Rondón and Catalan 2017, 2020). While numerous studies have investigated their responses to environmental changes, few have explored community patterns using metabarcoding techniques (Garcés‐Pastor et al. 2019). Most research has concentrated so far on species‐level representativeness and the use of diatoms in biomonitoring (Borrego‐Ramos et al. 2021; Pérez‐Burillo et al. 2022; Vidaković et al. 2024). Morphological species identification remains essential for validating metabarcoding studies. However, discrepancies between morphological identification and metabarcoding markers (Brown et al. 2022; Kim et al. 2024; Nistal‐García et al. 2021) have resulted in inaccuracies in species representation (Bailet et al. 2020; Wang et al. 2024), underscoring the necessity for more integrated approaches in diatom research. Due to this low taxonomic representation, Molecular Operational Taxonomic Units (MOTUs) are often used in community and ecological studies.

To address these gaps, this study aims to utilize metabarcoding to provide a comprehensive understanding of diatom communities in the Pyrenees mires. Mire ecosystems are great environmental heterogeneities that can support diverse diatoms (Gonzalez‐Saldias et al. 2025), being less studied than rivers or lakes. For this reason, the study of diatom communities in mires can provide relevant information on diversity and community patterns using universal markers. We hypothesized that genetic markers would provide accurate information on the diatom community patterns in high mountain mires. While alpha diversity describes local richness and diversity, community dissimilarity is a change between communities. To test this hypothesis, we employed two genetic markers, 18S rRNA and COI, and compared the results with morphological identification.

## 2 Methodology

### 2.1 Sample Drawing

**FIGURE 1:**
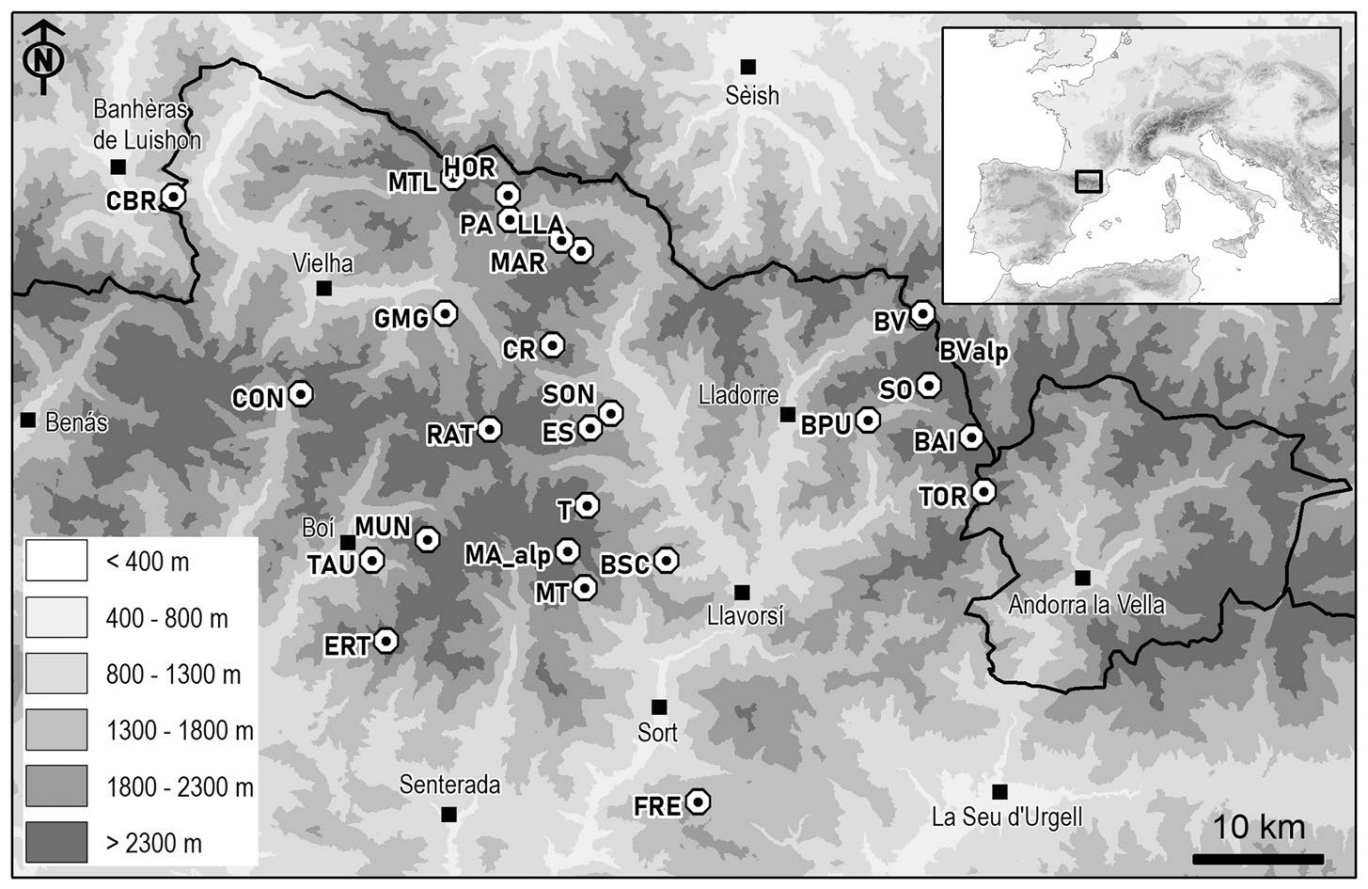
Geographical location of the studied mires. Black dots correspond to the mires sampled in the Catalan Pyrenees in the summer of 2018, and the gray triangle represents municipalities. Code, location name, and coordinate in Table S6.

A total of 66 plots on 26 mires located in the Central Pyrenees were sampled in the summers (July and August) of 2016, 2017, and 2018 under the LIFE+ Limnopirineus project (Figure 1). At each plot, five regularly spaced bites of topsoil (5 cm deep) samples (100 mL), including the bryophyte layer, were preserved in 96% ethanol. Half of each sample was allocated for morphological analysis, and the other half for molecular identification. The following variables were measured to characterize each sampling site: water table depth, vegetation cover, pH, and electrical conductivity (Table S1). The pH and electrical conductivity were measured using a WTW ProfiLine pH 3310 Portable pH Meter. We used a plot of 2.5 × 2.5 m to estimate vegetation cover. For each plot, we visually estimated the percentage cover of all plant species. We then classified the species into broad functional groups: vascular plants, bryophytes, sphagnum, pleurocarpic mosses, acrocarpic mosses, and liverworts (Marchantiophyta). To obtain the total cover of each functional group, we summed the percentage cover of the individual species within the group. Moreover, a sample of groundwater (100 mL) was taken at each plot, and the composition of metals and minerals (K, Mg, Mn, Na, S, and Si) was also analyzed by the Inductively Coupled Plasma (ICP‐OES) method. As for climatic regional variables, we used the Pyrenean Digital Climatic Atlas with a 30‐m resolution (Batalla et al. 2018). We retrieved mean annual precipitation, annual potential solar radiation, and growing degree days. The growing degree day value (GDD) for each site was estimated as follows:

$$\mathrm{GDD}=\sum \left(\left(\frac{\left(T_{\max}+T_{\min}\right)}{2}\right)-T_{\text{base}}\right)\times N$$

*T*_max_ is the day's maximum temperature, *T*_min_ is the day's minimum temperature, *T*_base_ equals zero, and *N* is the number of days/months.

### 2.2 Morphological Identification of Diatoms

Half of each sample taken was used for morphological identification of diatoms, and organic matter was removed by treating the samples with 33% w/v hydrogen peroxide for 24 h at 100°C, followed by an additional 24 h at room temperature to remove all organic matter. After this treatment, the supernatant was carefully discarded, and the samples were rinsed with distilled water. The cleaned diatom valves were then mounted on permanent microscope slides using Naphrax. Identification and enumeration of diatoms were conducted under a POLYVAR microscope with Differential Interference Contrast with an objective of 100× magnification and 1.3 numerical aperture, counting 500 valves per sample. Taxonomic identification followed the monographs of Krammer and Lange‐Bertalot (2004a, 2004b, 2004c, 2004d), Lange‐Bertalot (2000, 2001), Lange‐Bertalot et al. (2011, 2017), and Rivera‐Rondón and Catalan (2020). The relative abundance of each diatom species was calculated for subsequent analysis.

### 2.3 DNA Extraction

DNA extraction was performed at the genetic laboratories of the Department of Evolutionary Biology, Ecology, and Environmental Sciences at the University of Barcelona. All extraction procedures were conducted under a laminar‐flow cabinet to prevent potential contamination. Between DNA sample extractions, all the equipment was thoroughly cleaned with 10% sodium hypochlorite and rinsed in Milli‐Q water (Garcés‐Pastor et al. 2019).

Peat samples were homogenized using a 600 W hand blender. Each homogenized sample (0.3 g) was extracted using the Soil DNA Isolation Plus Kit (Norgen Biotech Corp., Ontario, Canada, www.norgenbiotek.com). An extraction blank consisting of 300 μL of molecular biology‐grade water was included in the DNA extraction batch, processed, and sequenced along with the rest of the samples (Garcés‐Pastor et al. 2019).

### 2.4 PCR and Sequencing

Two markers were used to identify a broad taxonomic range of eukaryotic taxa. The Leray‐XT primer set, which amplifies a 313 bp fragment of the mitochondrial COI marker (miCOIintF‐XT 5′‐GGWACW RGWTGRACWITITAYCCYCC‐3′; Wangensteen et al. 2018), and jgHCO2198 5′‐TAIACYTCIGGRT GICCRAARAAYCA‐3′ (Geller et al. 2013) were also used. Additionally, the V7 region of the nuclear‐encoded ribosomal 18S rRNA gene was amplified using the 18S_allshorts primers (100–110 bp, 5′‐TTTGTCTGSTTAATT SCG‐3′ and 5′‐TCACAGACCTGTTATTGC‐3′; Guardiola et al. 2015).

PCR products were pooled by marker and purified using Minelute columns. Two Illumina libraries were prepared from the DNA pools using the NEXTflex PCR‐free kit and tagged for identification. Both libraries were sequenced together on an Illumina MiSeq using v3 chemistry with 2 × 250 bp paired‐end reads. Bioinformatics analyses were conducted using the OBITools software suite (Boyer et al. 2016) following the methods described by Garcés‐Pastor et al. (2019). MOTUs' construction was conducted with SWARM 1.2.19 (Kopylova et al. 2016). For taxonomy annotation, representative MOTU sequences were BLAST (v2.9.0) against RSyst::diatom reference base v7 (Rimet et al. 2016) with an identity cut‐off and minimum alignment length of 80%. Singletons and samples with < 50 reads were removed from the analysis. Rarefaction curves indicated that sequencing depth approached saturation in the number of MOTUs for both markers at 53 reads and 103 reads per sample for 18S rRNA and COI, respectively.

### 2.5 Statistical Analysis

MOTUs were used to calculate richness, the Shannon index, and Pielou's evenness for each sample in the molecular dataset. To evaluate significant differences in Shannon, Pielou, and species richness between the molecular and morphological identification methods, Kruskal–Wallis tests were performed because the data did not meet assumptions of normality and homoscedasticity. To quantify the differences in alpha diversity (Richness, Shannon, and Pielou evenness) between the morphological and molecular identification techniques, log_2_‐fold changes were calculated. The log_2_ fold change is the log_2_ of the ratio between molecular and morphological alpha diversity. Then, positive log_2_‐fold changes indicate higher alpha diversity in molecular approaches related to the morphological‐derived estimations, while negative values indicate underestimation. To assess significant differences in alpha diversity (richness, Shannon, and Pielou) between morphological and molecular identification, we performed Student's *t*‐tests for two samples, or Wilcoxon tests when assumptions were not met.

Rarefaction was applied to the reads to homogenize the sequencing depth. Reads were rarefied to the read count of the lowest sample in each marker. We used the “rarefy” function from the vegan package to MOTU rarefaction. Bray–Curtis dissimilarity was calculated with square root‐transformed morphological abundance and molecular reads matrices to assess sample dissimilarities. The degree of correspondence between the ordinal results obtained for morphological and molecular datasets was evaluated with a Procrustean analysis followed by a PROTEST (Goodall 1991; Jackson 1995). Procrustean plots were generated from the ordinal results of morphological and molecular‐based multidimensional scaling (MDS) based on the Bray–Curtis distance matrixes. Distance‐based redundancy analyses (dbRDA) were performed on the Bray–Curtis dissimilarity matrix to elucidate the relationship between environmental variables and the diatom community composition. All statistical analyses were performed with MOTUs due to low taxonomic assignment.

All the statistical analyses were conducted in R (v4.2.3) using the packages vegan (Oksanen et al. 2022), dplyr (Wickham et al. 2023), and microbiome (Lahti and Shetty 2017).

## 3 Results

### 3.1 Environmental Variables

The high mountain mires studied displayed great variability within the mire ecological habitats (Table 1). The environmental variability related to altitudinal changes, such as temperature (1226–2967 GDD), and annual precipitation ranging from 943.6 to 1745.5 mm, presented a great range. The pH varied between 4.51 and 7.76, and K (1.480–21.769 mg/L) has a wide range. The vegetation cover of both vascular plants and bryophytes varied considerably. Within the bryophyte group, *Sphagnum* mosses and pleurocarpic mosses (0–119 and 0–94, respectively) presented a great cover range (Table 1).

**TABLE 1:** Mean and range of environmental variables in the Pyrenees mires. The sum of species covers the different groups of *Sphagnum*, Brown mosses, Acrocarp mosses, Liverworts, Total bryophytes, and Vascular plants.

Environmental variables	Mean	Range
GDD (°C)	1871.561	1226 to 2967
Precipitation (mm)	1337.882	943.6 to 1745.5
pH	5.977	4.51 to 7.76
WTD (cm)	−6.117	−36.6 to 0.0
Radiation (kJ/m^2^ × day)	1871.561	1546 to 2174
*Sphagnum*	12.038	0 to 119
Pleurocarpic mosses	25.879	0 to 94
Acrocarp mosses	4.849	0 to 52
Liverworts	0.144	0 to 2
Total bryophytes	44.909	0 to 119
Vascular plants	106.940	0.055 to 285
K (mg/L)	4.069	1.480 to 21.769
Mg (mg/L)	1.018	0.174 to 7.379
Mn (mg/L)	0.122	0.005 to 3.233
Na (mg/L)	2.020	0.431 to 9.652
P (mg/L)	0.178	0.050 to 0.951
S (mg/L)	2.167	0.221 to 9.821
Si (mg/L)	0.519	0.050 to 2.001

### 3.2 Morphological Identification

We identified a total of 383 species belonging to 65 genera using light microscopy (Table S2). The average number of species per sample was 42.33 ± 10.11. The most abundant species were represented by *Achanthidium minutissimum* (Kützing) Czarnecki, *Staurosira contruens* var. *venter* (Ehrenberg) P.B.Ham., *Eunotia incisa* W.Sm. ex W.Greg, *Eolimna minima* (Moser et al. 1998), and *Kobayasiella micropunctata* (H.Germ.) Lange‐Bert. 1999 contributed to > 1% of total individuals across samples (Table S3).

### 3.3 Diatom Molecular Identification

The DNA recovered from the peat samples yielded 4.9–31.2 ng/μL concentrations. For 18S rRNA sequences, 80,469 reads were retrieved (1219 ± 2772 reads per sample; mean ± SD). The final dataset for COI included a total of 153,315 reads (2322 ± 3419 reads per sample). The taxonomic assignment based on 18S rRNA resulted in a total of 166 MOTUs presented by 116 species belonging to 16 genera. In comparison, identification based on COI resulted in a total of 277 MOTUs corresponding to 18 species from six genera (Table S4). Taxonomic assignment at the genus and species level is 6.5% for COI and 69.9% for 18S rRNA (> 95% percentage identity). For each marker, more than 1% of the total abundance contribution was made by 26 MOTUs. MOTUs were present at the genus and species level (*n* = 2, > 95% identity, and *n* = 5, > 97% identity, respectively). Conversely, 18S rRNA presented a higher number of MOTUs at the species level (*n* = 21 at > 97% identity) and four at the genus level (> 95% identity). The 18S rRNA marker presents four species that are identical in morphology. The two most abundant species are *A. minutissimum* and *S. contruens*, but with lower relative abundance compared to morphological identification. Only one coincides with COI, but the level of identity does not exceed 90% (Table S3).

### 3.4 Morphological and Molecular Alpha Diversity

**FIGURE 2:**
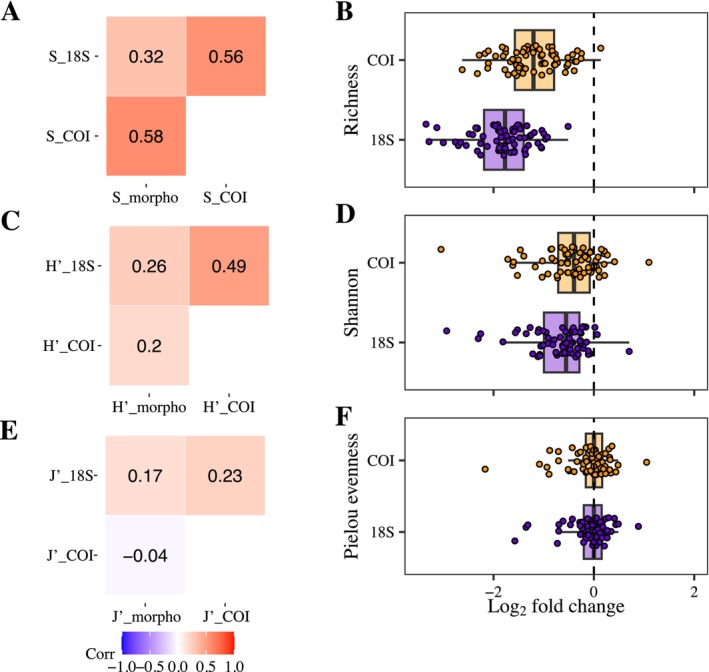
Spearman correlation between molecular and morphological identification in richness (A), Shannon index (C), and Pielou evenness (E). Comparison of alpha diversity indices in the three approaches to diatom communities of high mountain mires in richness (B), Shannon index (D), and Pielou evenness (F), molecular with morphological identification. Log_2_‐fold change shows the magnitude of differences between the morphological and molecular identification techniques in terms of alpha diversity, using the morphological‐derived indexes as a reference (dotted line). Positive log_2_‐fold changes indicate overestimation of alpha diversity in the molecular approaches compared to morphological, while negative values indicate underestimation.

Morphological diatom richness exhibited a positive and significant correlation with MOTUs richness for 18S rRNA (Spearman correlation 0.32, *p* < 0.01) and COI (Spearman correlation 0.58, *p* < 0.001), with COI‐based estimations showing the highest correlation (Figure 2A). However, COI and 18S rRNA underestimated diatom richness compared to morphological data (Student's *t*‐test, *p* < 0.001, Figure 2B). The Shannon index indicated that COI and 18S rRNA had a similar correlation with morphological identification (Figure 2C). Furthermore, COI demonstrated greater similarity to the morphological data compared to 18S rRNA, as evidenced by a log_2_‐fold change around 0 (Figure 2D). However, these relations were significantly when compared to morphological estimations (Wilcoxon test, *p* < 0.001). For Pielou's evenness, we observed a positive correlation only between morphological data and 18S rRNA. In contrast, a negative correlation was noted with COI (Figure 2E). In both instances, the evenness calculated using the markers was found to be similar to the obtained from the morphological data (Wilcoxon test, *p* > 0.05, Figure 2F).

### 3.5 Morphological and Molecular Diatom Dissimilarity

**FIGURE 3:**
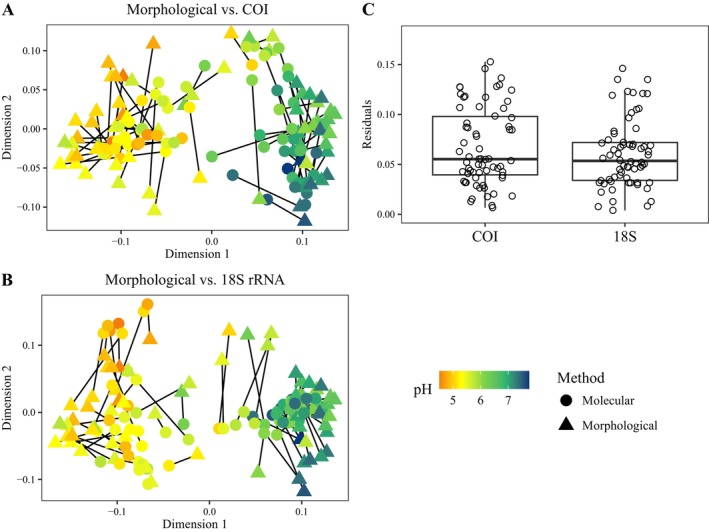
Comparison of MDS through Procrustes analysis between morphological versus COI (A) and morphological versus 18S rRNA (B). Residual values from comparing morphological identification and the two molecular identifications (18S rRNA and COI) (C).

Diatom communities have a high variability in species composition (Figure 3). Overall, Procrustes superimposition analysis showed a high similarity between morphological compositional variations and molecularly resolved communities for the sampled sites (*r* > 0.78, *p* < 0.001; Figure 3). Both molecular markers significantly represented the diatom community pairwise dissimilarities, with 18S rRNA holding the higher similitude with morphological assignations than COI (morphological‐COI PROTEST m1‐2 = 0.40, *r* = 0.78, *p* = 0.001; morphological‐18S PROTEST m1‐2 = 0.32, *r* = 0.83, *p* = 0.001) (Figure 3A,B). Despite the observed variability, the residuals from Procrustes analysis for COI and 18S rRNA were not significantly different (Student's *t*‐test, *p* = 0.207) (Figure 3C).

### 3.6 Community Drivers

The dbRDA analysis indicated that the environmental drivers of community composition were similar for the morphological and molecular methods (COI and 18S rRNA) (Table 2). The environmental variables explain over 27% of the variability in both methods. The dbRDA1 explains 75.56%, 61.28%, and 63.82% for morphological, COI, and 18S rRNA identifications, respectively. The dbRDA2 were similar for all identifications, with values of 10.64% for morphological identification, 17.35% for COI, and 11.90% for 18S rRNA. Among these drivers, pH was the main structuring variable, followed by temperature (GDD) and Mg concentrations (Table 2).

**TABLE 2:** Distance‐based redundancy analysis (dbRDA) with forward selection based on the Bray–Curtis dissimilarity matrix, with environmental variables related to local factors, and vegetation structure for morphological data, COI, and 18S rRNA.

Data	Environmental variables	Forward selection		
		*R* ^2^	*F*	*p*
Morphological	pH	0.150	11.329	0.001
	GDD	0.027	2.058	0.002
	Mg	0.029	0.264	0.001
	Precipitation	0.019	1.499	0.036
COI	pH	0.131	9.636	0.001
	GDD	0.043	3.241	0.001
	Mg	0.031	2.418	0.001
	*Sphagnum*	0.021	1.617	0.016
	Acrocarp mosses	0.018	1.420	0.046
18S rRNA	pH	0.162	12.395	0.001
	GDD	0.031	2.393	0.002
	Mg	0.034	2.700	0.001
	S	0.022	1.779	0.014
	Pleurocarpic mosses	0.020	1.695	0.019
	Al	0.021	1.674	0.019

**FIGURE 4:**
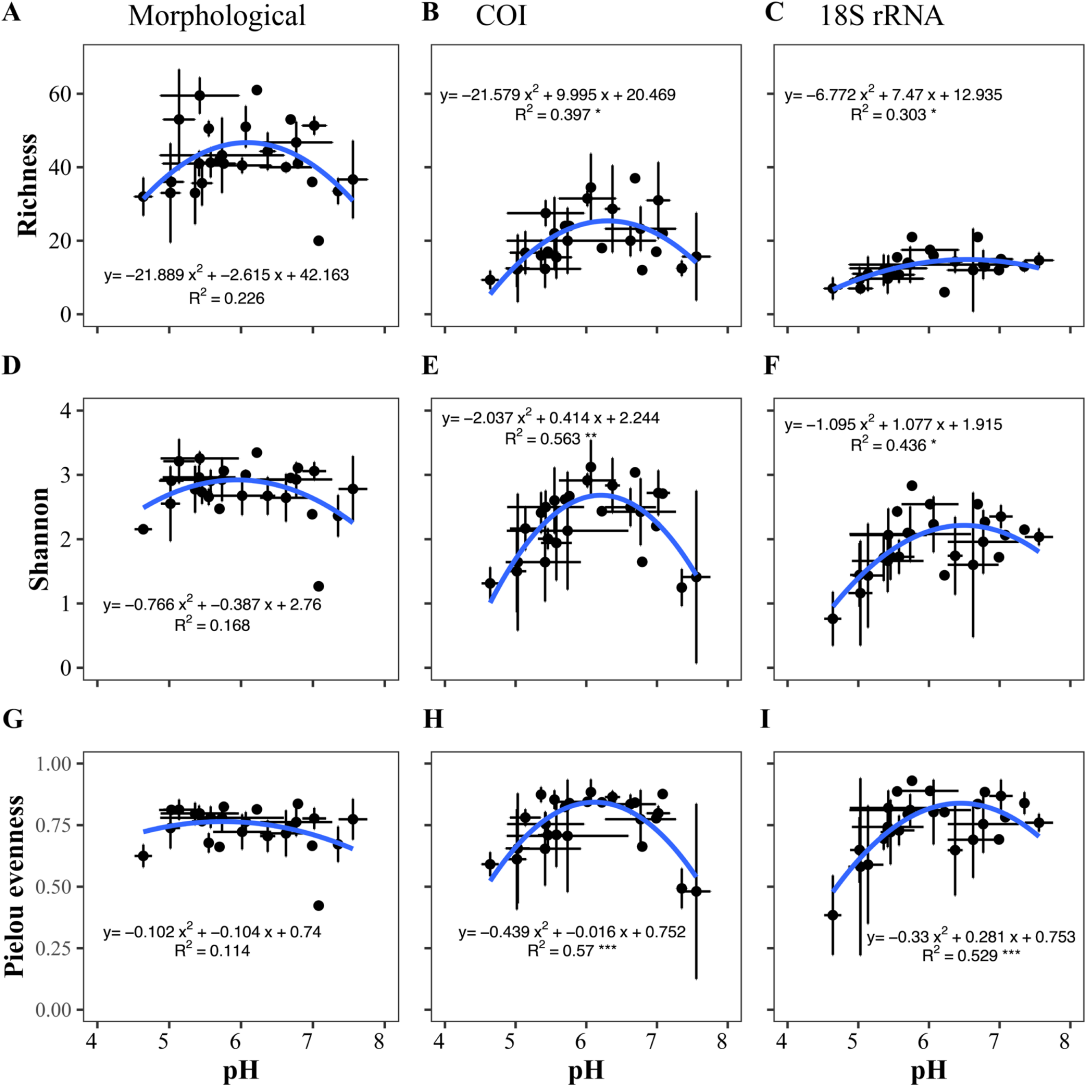
Correlation between pH and alpha diversity variables (Richness, Shannon index, and Pielou) through morphological (A, D, G), COI (B, E, H), and 18S rRNA (C, F, I) datasets ordered by increasing pH. The dot is the average pH versus the average of the corresponding alpha diversity variable of each locality. Lines are the standard deviations of pH on the horizontal axis and the corresponding alpha on the vertical axis. The blue line is an adjusted polynomial model of order two.

Diatom richness exhibited a clear pattern along the pH gradient for both morphological and molecular marker identifications (Figure 4A–C). Morphological identification showed higher richness (20–61 species), followed by COI (9–37 MOTUs), and 18S rRNA (6–21 MOTUs). Similarly, the Shannon diversity changes along pH were similar in the morphological and molecular data, ranging from 1.26 to 3.53 in morphological, 0.27–3.42 for COI, and 0.27 to 2.83 for 18S rRNA (Figure 4D–F). Pielou's evenness estimations were similar between morphological and molecular data, morphological data varied between 0.42 and 0.86, while COI and 18S rRNA presented higher variability in the values of equitability from 0.15 to 0.92 for COI and 0.20 to 0.93 for 18S rRNA (Figure 4G–I).

## 4 Discussion

COI and 18S rRNA markers showed a strong correspondence for diatom composition and response to environmental changes, despite not being specific markers for diatom identification. The most important variable structuring the diatom community was pH, which was observed in both morphological and molecular identification (Figures 3 and 4; Table 2). The importance of pH in diatom community structure has been reported previously, showing that it strongly influences diatom communities (Chen et al. 2014; Gonzalez‐Saldias et al. 2025; Hargan et al. 2015). Both genetic markers are good for detecting these patterns and environmental variables affecting diatom communities. The pH 5.5 and 6.5 are critical for diatom communities, causing compositional changes (Gonzalez‐Saldias et al. 2025; Hargan et al. 2015), which can be seen in our results (Figure 4).

### 4.1 Molecular Markers as Community Composition Indicators

Metabarcoding approaches for diatom identification at the community level provide information comparable to morphological‐based analysis. Our results show that COI and 18S rRNA, both universal markers, are robust for detecting community (dis)similarities (b‐diversity patterns). This is supported by the strong correlation in the Procrustean analysis along the environmental variables, in contrast to similar studies conducted in other aquatic habitats, such as ponds and rivers, where such correspondence is rarely observed (Brown et al. 2022; Nistal‐García et al. 2021; Vasselon et al. 2019). These results highlight the potential of utilizing universal markers in facilitating the extensive labor associated with diatom identification, particularly in heterogeneous habitats with high biodiversity and rare species (Gonzalez‐Saldias et al. 2025).

### 4.2 Taxonomic Identification

Poor taxonomic assignment is a recurrent issue in diatom molecular identification (Rivera et al. 2018; Vasselon et al. 2017). We found that 62.16% of the total reads in 18S rRNA and only 4.97% in COI were assigned to the species level. Both markers underestimated species richness, a 2 and 4‐fold for COI and 18S rRNA, respectively (Figure 3). Comparatively, 18S rRNA was more taxonomically accurate than COI. This finding contrasts with similar studies, which have generally reported that molecular identification tends to overestimate species richness relative to morphological assessments in rivers (Mora et al. 2019; Tapolczai et al. 2019; Wang et al. 2024). By contrast, a smaller number of taxa were identified using molecular techniques in less studied environments, such as glaciers (Chamorro et al. 2024). The poor taxonomic assignment may be related to the lack of studies on mire diatoms and, therefore, a poor representation of these species in the reference databases.

There were differences in the specificity of mitochondrial and ribosomal universal markers in Bacillariophyta. Our results indicate that the low species diversity captured by COI is due to the poor dataset and its poor performance in discriminating abundant and widespread diatom genera in mires, such as *Nitzschia*, *Eunotia*, and *Pinnularia* (Table S5), consistent with previous research (Hamsher et al. 2011; Moniz and Kaczmarska 2009; Trobajo et al. 2010). However, COI can effectively identify species affiliated with the *Sellaphora* genus, and the *Gomphonema parvulum* species, a recurrent dominant species in running waters (Evans et al. 2008, 2007; Mann et al. 2010). The V7 region of 18S rRNA has been successfully used to identify marine and mire eukaryotic organisms (Garcés‐Pastor et al. 2019; Guardiola et al. 2015). However, the V7 region in our study showed limited resolution in richness and Shannon index (Figure 2). Some studies suggest the V4 region is a better alternative, as it encompasses numerous hypervariable regions, including inversions, insertions, and deletions, making it more suitable for diatom species identification (Alverson et al. 2006; Zimmermann et al. 2011).

Universal markers are aimed at identifying most of the total diversity of different groups of organisms within ecosystems. Markers such as COI and 18S rRNA allow the study of eukaryotic heterotrophs and autotrophs, which vary in size and lifestyle. However, both markers have several limitations when it comes to taxonomic identification. First, the 18S rRNA is characterized by conserved regions with limited polymorphisms and, consequently, is more prone to taxonomic inaccuracies (Kermarrec et al. 2013). While COI has been extensively studied in metazoans (Che et al. 2012; Elbrecht and Leese 2017; Sultana et al. 2018), its efficiency in resolving photosynthetic organisms such as diatoms in databases remains poor (Kermarrec et al. 2013; Trobajo et al. 2010). Second, the poor taxonomic coverage can be attributed to the limited diatom database size available for COI (293 sequences) and 18S rRNA (2646 sequences) in RSyst::diatom reference base v7 compared to for example bacterial databases, such as the SILVA 16S small subunit database, which contains approximately 500,000 representative sequences (Quast et al. 2013; Rimet et al. 2016). Furthermore, the species examined in this study originated from highly heterogeneous environments, making them difficult to cultivate and, consequently, underrepresented in public archives. An alternative to address this issue is using diatom‐specific markers, such as the Rubisco *rbc*L (Kulaš et al. 2022). Therefore, COI and 18S rRNA appeared as not suitable identification markers for studies of biodiversity, because both underestimate alpha diversity (i.e., Richness and Shannon index), leading to data misinterpretation due to, for example, the poor coverage of the abundant representative genera in mires (Table S5).

### 4.3 Challenges in Molecular Identification for Diatoms

This study observed differences in the taxonomic assignment and number of MOTUs identified for the two markers used. These differences may be due to species‐specific gene copies, as observed in other studies with rbcL and 18S rRNA markers, where the copy number of different species is related to biovolume and therefore needs to be corrected (Kermarrec et al. 2013; Vasselon et al. 2019). Considering such a correction factor would help to improve the estimation of dominancy and abundance to match morphological‐based estimations (Godhe et al. 2008; Vasselon et al. 2018). In addition, the presence of cryptic species, environmental DNA, and degraded DNA can also bias the taxonomic resolution (Vasselon et al. 2018).

The variety of pipelines used for metabarcoding sequencing processing can generate differences in downstream analyses (Bailet et al. 2020). The bioinformatic analysis encompasses various processes, often including sequence primer clipping, quality check, and trimming, which may introduce variability in the taxonomic assignment and community representativeness (Bailet et al. 2020; Mora et al. 2019; Zimmermann et al. 2015). Another common issue in environmental DNA studies is the sequencing depth achieved with universal markers and untargeted organisms, which may affect the number of reads and MOTUs, directly impacting alpha diversity indices, as may be the case in our study.

## 5 Conclusion

In conclusion, the universal markers COI and 18S rRNA showed a high correspondence for diatom beta diversity and its response to environmental changes. Taxonomic identification through metabarcoding represents a viable alternative for the study of diatom community patterns. This approach can reduce the time required for sample identification and data processing compared to traditional methods. However, these molecular markers are unsuitable for precise taxonomic assignment and diversity studies because they underestimate alpha diversity, leading to misinterpretation of data.

## Author Contributions

**Fernanda Gonzalez‐Saldias:** conceptualization (equal), data curation (lead), formal analysis (lead), funding acquisition (lead), investigation (equal), methodology (equal), software (lead), visualization (lead), writing – original draft (lead), writing – review and editing (lead). **Joan Gomà:** conceptualization (equal), funding acquisition (supporting), supervision (lead), writing – review and editing (equal). **Sandra Garcés‐Pastor:** conceptualization (equal), resources (supporting), writing – review and editing (equal). **Owen S. Wangensteen:** methodology (equal), resources (supporting), software (equal), writing – review and editing (supporting). **Albert Pèlachs:** conceptualization (equal), resources (supporting), writing – review and editing (equal). **Aaron Pérez‐Haase:** conceptualization (equal), funding acquisition (lead), investigation (equal), methodology (lead), project administration (lead), resources (lead), supervision (lead), writing – review and editing (lead).

## Funding

F.G.‐S. was funded by the National Agency for Research and Development (ANID)/Scholarship Program/DOCTORADO BECAS CHILE/2020–72210270. This work has been supported by coordinated project grants from: (i) the Ministerio de Economia y Competitividad (Spanish Ministry of Economics and Competitiveness): “Calibracion de indicadores de influencia humana y climatica para la (re)interpretacion de la expansion postglacial y de las dinamicas forestales en los ultimos 18.000 anos” (PID2019108282GB‐I00/AEI/10.13039/501100011033); “Estudio biogeografico historico comparado (Montaña Cantabrica, Sistema Central y Pirineos): 18000 años de cambios climáticos y antrópicos sobre especies forestales indicadoras” (CSO2015‐65216‐C2‐1‐P); (ii) Organismo Autónomo de Parques Nacionales (OAPN) under the project BIOOCULT (OAPN 2413/2017); (iii) European Union LIFE programme under the project LIFE Limnopirineus (LIFE13 NAT/ES/001210) and the LIFE Resque Alpyr (LIFE20 NAT/ES/000369). S.G.‐P. was also supported by the Beatriu de Pinós Programme (BP‐2021‐00131) and a fellowship from “la Caixa” Foundation (ID 100010434, fellowship code LCF/BQ/PI24/12040011). O.W. was also supported by the Blue DNA project (PID2023‐1466307OB).

## Conflicts of Interest

The authors declare no conflicts of interest.

## Supporting information


**Tables S1–S6:** ece372644‐sup‐0001‐TablesS1‐S6.zip.

## Data Availability

Sequence data used in this study are deposited in the European Nucleotide Archive (accession number PRJEB87341). The relative abundance data (Table S2), metadata (Table S1), percentage of identity of molecular identification (Table S4), and code will be available in GitHub: https://github.com/fergonzalezsaldias/diatom.morphology.dna.
