# Supplementary material for: A Comparative Analysis of DNA Metabarcoding and Morphological Identification in Diatoms Reveals Similar Patterns of Environmental Response
Source: Ecol Evol. 2026 Feb 17;16(2):e72644. doi: 10.1002/ece3.72644 (PMC12912883; doi:10.1002/ece3.72644)
Supplement: Supplementary file 1 — Tables S1–S6: ece372644‐sup‐0001‐TablesS1‐S6.zip. [file ECE3-16-e72644-s001.zip › TablesS3,S5,S6.docx]

**Supplementary material**

**Table S1:** Environmental variables in each location of the mires sampled. The water table depth, vegetation cover, pH, electrical conductivity, and composition of minerals and metals.

**Table S2:** Relative abundances of diatoms in morphological and molecular (COI and 18S rRNA rRNA MOTUs) identification.

**Table S3:** The most abundant species of diatoms found in the sampled mires in morphological and molecular identification (only those with >1% relative abundances; mean ± standard deviation in morphological and mean in molecular). The percentage of the total individuals for morphological and molecular identification, the percentage of identity is related to the taxonomic assignation in the molecular method.

| **Morphological** | | **COI** | | | | **18S rRNA** | | | |
| --- | --- | --- | --- | --- | --- | --- | --- | --- | --- |
| **Species** | **Relative abundances (%)** | **MOTUs** | **Relative abundances (%)** | **Taxonomic assignation** | **Percent**  **identity** | **MOTUs** | **Relative abundances (%)** | **Taxonomic**  **assignation** | **Percent**  **identity** |
| *Achnanthidium minutisimum* (Kütz.) Czarnecki 1994 | 11,48 ± 12,85 | BOGC_000264231 | 8.546 | *Pinnularia brebissonii* | 82.414 | BOGS_000014580 | 13.694 | *Navicula veneta* | 100 |
| *Staurosira construens* var. *venter* (Ehrenb.) P.B. Hamilton 1992 | 4,65 ± 7,33 | BOGC_000970727 | 5.002 | *Fistulifera saprophila* | 82.986 | BOGS_000134630 | 9.949 | *Eunotia glacialis* | 99.038 |
| *Eunotia incisa* W.Sm. ex W.Greg. 1854 | 4,47 ± 8,46 | BOGC_000258122 | 4.369 | *Nitzschia acidoclinata* | 82.993 | BOGS_000199997 | 9.034 | *Pinnularia neglectiformis* | 98.02 |
| *Eolimna minima* (Grunow) Lange-Bertalot 1998 | 4,08 ± 6,65 | BOGC_002889770 | 4.369 | *Pinnularia sp* | 83.333 | BOGS_000126337 | 4.974 | *Gomphonema parvulum* | 100 |
| *Kobayasiella micropunctata* (H.Germain) Lange-Bertalot 1999 | 4,00 ± 9,15 | BOGC_000654903 | 4.369 | *Pinnularia viridiformis* | 95.05 | BOGS_000172574 | 4.403 | *Achnanthidium minutissimum* | 100 |
| *Brachysira brebissonii* R.Ross in B.Hartley 1986 | 2,53 ± 4,56 | BOGC_001062949 | 3.722 | *Sellaphora minima* | 100 | BOGS_000124963 | 2.916 | *Frustulia crassinervia/saxonica complex* | 100 |
| *Caloneis tenuis* (W.Gregory) Krammer 1985 | 2,12 ± 3,87 | BOGC_002873710 | 2.648 | *Pinnularia viridiformis* | 84.746 | BOGS_000173013 | 2.544 | *Caloneis silicula* | 100 |
| *Frustulia saxonica* Rabenh. 1850 | 2,07 ± 5,46 | BOGC_000363872 | 2.471 | *Gomphonema parvulum* | 90.096 | BOGS_000180056 | 2.201 | *Pinnularia viridiformis* | 97.087 |
| *Navicula exilis* Kützing 1844 | 1,98 ± 4,48 | BOGC_001655856 | 2.148 | *Sellaphora pupula* | 86.306 | BOGS_000125502 | 1.944 | *Planothidium victori* | 94.175 |
| *Gomphonema parvulum* (Kützing) Kützing 1849 | 1,65 ± 3,07 | BOGC_001330265 | 2.030 | *Pinnularia subanglica* | 83.226 | BOGS_000125634 | 1.915 | *Neidium productum* | 99.02 |
| *Brachysira neoexilis* Lange-Bertalot 1994 | 1,62 ± 4,55 | BOGC_001655701 | 1.971 | *Nitzschia acidoclinata* | 99.681 | BOGS_000172272 | 1.915 | *Eunotia minor* | 99.038 |
| *Tabellaria flocculosa* (Roth) Kütz. 1844 | 1,60 ± 3,74 | BOGC_000462072 | 1.957 | *Sellaphora pupula* | 84.775 | BOGS_000167353 | 1.801 | *Pinnularia divergens* | 100 |
| *Meridion circulare* (Grev.) C.Agardh 1831 | 1,36 ± 7,67 | BOGC_000361410 | 1.927 | *Pinnularia sp* | 90.323 | BOGS_000290336 | 1.744 | *Pinnularia cf. isselana* | 100 |
| *Gomphonema gracilis* Ehrenberg 1838 | 1,35 ± 2,42 | BOGC_002120333 | 1.839 | *Ulnaria ulna* | 99.681 | BOGS_000158824 | 1.601 | *Pinnularia subgibba* | 99.029 |
| *Encyonema neogracile* Krammer 1997 | 1,17 ± 1,97 | BOGC_000948651 | 1.824 | *Sellaphora pupula* | 84.365 | BOGS_000133943 | 1.572 | *Pinnularia subgibba* | 96.117 |
| *Eunotia fallax* Cleve 1895 | 1,16 ± 3,81 | BOGC_000684921 | 1.809 | *Sellaphora minima* | 99.681 | BOGS_000458031 | 1.401 | *Amphora waldeniana* | 95.098 |
|  |  | BOGC_000020776 | 1.751 | *Nitzschia frustulum* | 83.388 | BOGS_000246536 | 1.372 | *Cymbopleura naviculiformis* | 96.154 |
|  |  | BOGC_000639602 | 1.545 | *Nitzschia acidoclinata* | 90.705 | BOGS_000150046 | 1.315 | *Pinnularia neglectiformis* | 97.087 |
|  |  | BOGC_000550512 | 1.530 | *Pinnularia viridiformis* | 95.71 | BOGS_000206295 | 1.287 | *Staurosira construens* | 100 |
|  |  | BOGC_000259233 | 1.324 | *Pinnularia cf. isselana* | 85.113 | BOGS_000179838 | 1.258 | *Navicula cryptocephala* | 99.048 |
|  |  | BOGC_002659394 | 1.250 | *Pinnularia acrosphaeria* | 86.174 | BOGS_000188077 | 1.144 | *Gomphonema micropus* | 100 |
|  |  | BOGC_003326158 | 1.133 | *Pinnularia cf. isselana* | 98.71 | BOGS_000133948 | 1.115 | *Lemnicola hungarica* | 95.146 |
|  |  | BOGC_002956160 | 1.103 | *Pinnularia acrosphaeria* | 86.538 | BOGS_000178967 | 1.058 | *Encyonopsis sp* | 98.058 |
|  |  | BOGC_000259352 | 1.089 | *Gomphonema parvulum* | 85.577 | BOGS_000172410 | 1.029 | *Gomphonema acuminatum* | 100 |
|  |  | BOGC_000020780 | 1.030 | *Nitzschia recta* | 80 | BOGS_000176094 | 1.029 | *Pinnularia subcommutata* var. *nonfasciata* | 100 |
|  |  | BOGC_000947790 | 1.015 | *Navicula capitatoradiata* | 91.054 | BOGS_000133951 | 1.001 | *Pinnularia neglectiformis* | 99.029 |

**Table S4:** Percentage of identity for COI and 18S rRNA. The percentage >97% represents species, and >95% is a genus identification.

**Table S5:** Diatom genera identified in the sampled mires of the Pyrenees with relative abundances greater than 1% and number of species for each one.

| **Genus** | **Number of species** |
| --- | --- |
| *Eunotia* Ehrenb. 1837 | 43 |
| *Pinnularia* Ehrenb. 1843 | 40 |
| *Nitzschia* Hass. 1845 | 24 |
| *Gomphonema* Ehrenb. 1832 | 23 |
| *Navicula* Bory de Saint-Vincent 1822 | 19 |
| *Encyonema* Kütz. 1833 | 18 |
| *Cymbopleura* Krammer 1997 | 13 |
| *Fragilaria* Lyngbye 1819 | 13 |
| *Achnanthidium* Kütz. 1844 | 10 |
| *Aulacoseira* Thwaites 1848 | 10 |
| *Caloneis* Cleve 1894 | 9 |
| *Encyonopsis* Krammer 1997 | 9 |
| *Chamaepinnularia* Lange-Bert. and Metzeltin 1996 | 7 |
| *Diploneis* Cleve 1894 | 7 |
| *Brachysira* Kütz. 1836 | 5 |
| *Eolimna* Lange-Bert. and Schiller 1997 | 4 |
| *Frustulia* Rabenhorst 1853 | 3 |
| *Meridion* Agardh 1824 | 3 |
| *Staurosira* Ehren. 1842 | 3 |
| *Tabellaria* Ehrenberg ex Kützing 1844 | 3 |
| *Kobayasiella* Lange-Bertalot 1999 | 2 |
| *Rhopalodia* O. Muller 1895 | 2 |

**Table S6:** Code, location name, and coordinates of the sampled area.

| **Code** | **Location name** | **Coordinates** | |
| --- | --- | --- | --- |
|  |  | **Longitude** | **Latitude** |
| BAI | Baiau | 1.422 | 42.607 |
| BPU | Bordes de Puntanou | 1.323 | 42.617 |
| BSC | Baiasca | 1.134 | 42.516 |
| BV | Pla de Boavi | 1.372 | 42.691 |
| BValp | Pla de Boavi | 1.373 | 42.693 |
| CBR | Clòt des Bruishes | 0.656 | 42.763 |
| CON | Conangles | 0.782 | 42.627 |
| CR | Clots de Rialba | 1.020 | 42.665 |
| ERT | Erta | 0.869 | 42.454 |
| ES | Estanyeres | 1.059 | 42.608 |
| FRE | Freixa | 1.169 | 42.347 |
| GMG | Barratge d'Aiguamòg | 0.918 | 42.685 |
| HOR | Horcalh | 0.975 | 42.770 |
| LLA | Llançanes | 1.046 | 42.732 |
| MA_alp | Mainera | 1.040 | 42.521 |
| MAR | Marimanha | 1.027 | 42.739 |
| MT | Montsent de Pallars | 1.057 | 42.495 |
| MTL | Montoliu | 0.922 | 42.782 |
| MUN | Muntanyó de Llats | 0.906 | 42.526 |
| PA | Parros | 0.977 | 42.753 |
| RAT | Ratera | 0.963 | 42.605 |
| SO | Sotllo | 1.381 | 42.643 |
| SON | Son | 1.078 | 42.618 |
| T | Trescuro | 1.057 | 42.553 |
| TAU | Taüll | 0.854 | 42.511 |
| TOR | Tor | 1.434 | 42.568 |
